# Effectiveness of first-line antiretroviral therapy and correlates of longitudinal changes in CD4 and viral load among HIV-infected children in Ghana

**DOI:** 10.1186/1471-2334-13-476

**Published:** 2013-10-13

**Authors:** Oliver Barry, Jonathan Powell, Lorna Renner, Evelyn Y Bonney, Meghan Prin, William Ampofo, Jonas Kusah, Bamenla Goka, Kwamena WC Sagoe, Veronika Shabanova, Elijah Paintsil

**Affiliations:** 1 From the Columbia University, New York, NY, USA; 2 Weill Cornell Medical College, New York, NY, USA; 3 University of Ghana Medical School, Accra, Ghana; 4 Noguchi Memorial Institute of Medical Research, University of Ghana, Accra, Ghana; 5 Yale Center for Analytical Sciences, Yale School of Medicine, New Haven, CT, USA; 6 Departments of Pediatrics and Pharmacology, Yale School of Medicine, New Haven, CT, USA

**Keywords:** Pediatrics, Antiretroviral therapy, Laboratory monitoring, Virologic failure, HIV drug resistance mutations

## Abstract

**Background:**

Antiretroviral therapy (ART) scale-up in resource-limited countries, with limited capacity for CD4 and HIV viral load monitoring, presents a unique challenge. We determined the effectiveness of first-line ART in a real world pediatric HIV clinic and explored associations between readily obtainable patient data and the trajectories of change in CD4 count and HIV viral load.

**Methods:**

We performed a longitudinal study of a cohort of HIV-infected children initiating ART at the Korle-Bu Teaching Hospital Pediatric HIV clinic in Accra, Ghana, aged 0-13 years from 2009-2012. CD4 and viral load testing were done every 4 to 6 months and genotypic resistance testing was performed for children failing therapy. A mixed linear modeling approach, combining fixed and random subject effects, was employed for data analysis.

**Results:**

Ninety HIV-infected children aged 0 to 13 years initiating ART were enrolled. The effectiveness of first-line regimen among study participants was 83.3%, based on WHO criteria for virologic failure. Fifteen of the 90 (16.7%) children met the criteria for virologic treatment failure after at least 24 weeks on ART. Sixty-seven percent virologic failures harbored viruses with ≥ 1 drug resistant mutations (DRMs); M184V/K103N was the predominant resistance pathway. Age at initiation of therapy, child’s gender, having a parent as a primary care giver, severity of illness, and type of regimen were associated with treatment outcomes.

**Conclusions:**

First-line ART regimens were effective and well tolerated. We identified predictors of the trajectories of change in CD4 and viral load to inform targeted laboratory monitoring of ART among HIV-infected children in resource-limited countries.

## Background

There is an unprecedented global effort to scale-up antiretroviral treatment (ART) and provide universal access to HIV care to all HIV-infected individuals. In resource-limited settings, outcomes of pediatric HIV treatment have been comparable to outcomes in resource-rich countries [1,2]. Several studies from sub-Saharan Africa have reported the effectiveness of ART using mortality as an outcome among HIV-infected children [2-5]. Moreover, children receiving ART in resource-limited settings have demonstrated low levels of drug toxicity, with rates of adherence and retention similar to those in resource-rich settings [3,4,6-8].

The success story of ART scale-up in resource-limited countries presents a unique challenge with regard to laboratory monitoring of treatment. The therapeutic benefits of ART are often limited by long-term toxicities and evolution of drug-resistant virus [9-11]. In resource-rich countries, HIV treatment is monitored routinely with laboratory measures such as blood chemistry, HIV viral load, and CD4 count for early detection of side effects of medications and drug-resistant virus [12,13]. Due to the lack of accessible and affordable laboratory services, routine laboratory monitoring is not feasible in most resource-limited countries [14]. Without laboratory monitoring, many patients may experience prolonged virologic failure and develop drug resistance mutations, which could ultimately limit second-line treatment options, increase morbidity, mortality and increase transmission of resistant viruses in the population [5,15,16]. The World Health Organization (WHO) recommends CD4 count monitoring every six months and viral load testing only when the capacity exists. [17]. There are limited data on laboratory monitoring of treatment from real world pediatric HIV clinics in resource-limited countries where there are frequent shortages of laboratory reagents, breakdown of equipment in addition to poor compliance with clinic appointments making testing at fixed intervals impossible.

We recently reported that a targeted approach based on predictors of CD4 recovery can be a viable and cost-effective way of monitoring ART in HIV-infected children in resource-limited settings [18]. To validate this finding, we conducted a prospective observational longitudinal study at Korle-Bu Teaching Hospital Pediatric HIV Clinic. The main objectives of the current study were: (1) to determine the real world effectiveness of first line ART and pediatric care utilization; and (2) to explore associations between readily obtainable data and the trajectories of change in CD4 count and HIV viral load.

## Methods

### Study population

This was a single-center prospective observational longitudinal study of HIV-infected children initiating ART at the Pediatric HIV/AIDS Care program at Korle-Bu Teaching Hospital in Accra, Ghana, from October 2009 to September 2012. The program currently cares for over 1,100 children. The first-line regimen available at the clinic is non-nucleoside analog (NNRTI)-based ART consisting of zidovudine (ZDV) plus lamivudine (3TC), plus either nevirapine (NVP) or efavirenz (EFV). Written consents from parents or guardians and assents from children were obtained before enrollment in the study. The study was reviewed and approved by the Ethics and Protocol Review Committees of University of Ghana Medical School and Yale School of Medicine.

At the time of enrollment, participants and guardians were interviewed to collect demographic and background information and their medical records were reviewed. The participants were seen and examined at the pediatric HIV clinic every 4-6 months, and more frequently as necessary, in accordance with local standard-of-care. CD4 cell count/percentage and HIV viral load determinations were done every 4-6 months. Adherence was determined by caregivers report and categorized according to WHO guidelines: adherence is said to be “good adherence” (i.e., missing ≤3 doses in a month, ≥95%), “fair adherence” (i.e., missing 4-8 doses in a month, 85%-94%), and “poor adherence” (i.e., missing ≥9 doses in a month, <85%) [19]. The study’s main outcome variables were CD4 absolute count, CD4 percentage and viral load over the study period.

### WHO definitions for treatment failure in HIV-infected children on ART

Clinical failure is defined as the appearance or reappearance of WHO clinical stage 3 or stage 4 events after at least 24 weeks on ART in a treatment-adherent child. Immunological failure is defined as developing or returning to the following age-related immunological thresholds after at least 24 weeks on ART, in a treatment-adherent child: (1) CD4 count of <200 cells/μL or percent CD4 <10% for a child ≥2 years to <5 years of age, (2) CD4 count of <100 cells/ μL for a child 5 years of age or older. Virologic failure is defined as a persistent HIV viral load of ≥ 5,000 copies/ml, after at least 24 weeks on ART, in a treatment-adherent child [19]. Based on the definitions of treatment failure above, ART treatment is deemed effective if none of the clinical, immunological or virologic criteria for failure are met after at least 24 weeks on ART. However, for purposes of this study, the definition of effectiveness is based on the virologic criteria for failure because it the most sensitive. Therefore treatment effectiveness was defined as HIV viral load <5,000 copies/ml after at least 24 weeks on ART.

### Other study measure

### CD4 cell count and HIV viral load measurements

CD4 absolute cell count and cell percentage were quantified by a dual-platform flow cytometry technology using a FACSCount system (Becton-Dickinson, Franklin Lakes, NJ, USA) at the clinical laboratory at Korle-Bu Teaching Hospital (KBTH) according to manufacturer’s instructions. The HIV RNA viral load testing was performed using the COBAS ® AMPLICOR Monitor test (Roche Diagnostic Systems, Branchburg, NJ, USA). The limit of HIV-1 RNA detection was 50 copies/mL. The laboratory is certified by the South African Public Health Reference Laboratory and participates in an external quality assurance testing program by the South African Public Health Reference Laboratory.

### HIV sequencing and mutational analyses

HIV genotyping was performed at Noguchi Memorial Institute of Medical Research (NMIMR), University of Ghana, using an in-house reverse transcriptase polymerase chain reaction (PCR) protocol. The reverse transcriptase (RT) and protease (PR)-coding regions were amplified in separate reactions using gene-specific primers and one-step RT-PCR techniques previously published [20]. The sequence data were assembled and edited using SeqMan (DNASTAR, USA). The FASTA-formatted sequences were then submitted online to the Stanford University HIVDR Database (http://hivdb.stanford.edu) to generate a mutation list and subtype information. The resulting mutation list was compared with the WHO mutation lists for HIVDR surveillance [21,22]. HIV-1 drug susceptibility of participants’ genotype was predicted using the Stanford algorithm, version 6.1.0 (http://hivdb.stanford.edu).

### Statistical analysis

Descriptive statistics, such as counts, percents and quartiles were used to summarize characteristics of children in the study. CD4 absolute counts and viral loads were log-transformed prior to statistical modeling. Linear mixed effects modeling was the primary statistical analysis. Along with such fixed effects as child’s gender, primary caregiver status, ART regimen and WHO HIV clinical staging, a random intercept with unstructured covariance was included. This allowed each child to have his/her specific constant variation around the group means. Intra-class correlation (ICC) for each outcome of interest was calculated to quantify the variability in the observed data due to individual child.

Longitudinal analyses were performed using mixed models to examine differences in CD4 cell count, CD4 percentage and HIV viral load by gender, WHO clinical staging and other demographic characteristics. Mixed model analysis was used as this approach does not require all participants to have the same number of measurements and uses all available outcome data, thus providing an unbiased estimate of the model parameters, given that missing outcomes are missing at random (MAR). Under the MAR assumption we expect that the missing outcomes are only dependent on the observed data and not on the unobserved data [23]. Along with the fixed linear and quadratic terms for time, a random subject-specific intercept, and an initial covariance set at Compound Symmetry (CS), each fixed factor was tested independently, with or without its interaction with the time terms. The best covariance structure was determined using the likelihood ratio test or Akaike Information Criterion (AIC), as appropriate. Findings are presented as parameter estimates from the mixed effects models, interpretable as the change in the average level of the outcome of interest (conditional on the random effect) for every 1 unit increase in the predictor variable for continuous predictors, or as the difference in the outcome of interest between a level of a predictor and a reference level/group for categorical variables. Analyses were performed using SAS 9.2 (Cary, NC).

## Results

### Baseline characteristics of study population

Ninety HIV-infected children aged 0 to 13 years initiating ART were enrolled to the study between October 2009 and September 2012. The number of available visits ranged between 1 and 9, with a median of 4 visits. This corresponded to a median of 16 months of follow-up (ranging from 0 to 32 months). Table 1 summarizes demographic and clinical characteristics of study participants. There were 53 males (59%), with over half of the children still living with their biologic parents (66.3%). Over half of children started ART at 5 years old or older and more than a quarter of the participants had a previous tuberculosis (TB) diagnosis.

**Table 1:** Characteristics of study population; N = 90 children

**Characteristic (categorical variables)**	**Summary Statistics, N (%)**				
Gender					
Male	53 (58.9)				
Female	37 (41.1)				
Primary Caregiver					
Parent	59 (66.3)				
Other	30 (33.7)				
Age at ART Initiation					
< 2 years	14 (15.6%)				
[2-5) years	25 (27.8%)				
≥ 5 years	51 (56.7%)				
ART Regimen					
3TC, ZDV, EFV	66 (73.3)				
3TC, ZDV, NVP	18 (20.0)				
Other	6 (6.7)				
WHO HIV Clinical Stage					
I	6 (7.2)				
II	14 (16.9)				
III	32 (38.9)				
IV	31 (37.4)				
Previous TB Diagnosis					
Negative	56 (62.2)				
Positive	34 (37.8)				
Characteristics (continuous variables)	Summary Statistics				
	Min,	Max,	Q1,	Median	Q3
Age at ART Initiation (months)	1.0	153.6	44.4	70.6	107.2
Number of Available Visits During Study Period	1	9	3	4	6
Time Between ART Initiation and First Available Visit (months)	0.0	8.5	0.4	1.4	3.0
Time Between ART Initiation and Last Available Visit (months)	0.0	32.0	10.0	16.0	21.0
CD4 Labs at First Available Visit Post ART Initiation					
Absolute Count (cells/μL)	9.0	3,293	339.0	663.0	1,075
Log(Absolute Count)	2.2	8.1	5.8	6.5	7.0
Percentile	0.0	50.5	10.3	16.9	26.6
Viral Load at First Available Visit Post ART Initiation					
Viral Load (Copies/mL)	63.0	3.6 × 10^6^	400.0	463.0	10,600
Log (Viral Load)	4.1	15.1	6.0	6.1	9.3

Abbreviations: *ART* Antiretroviral Therapy, *3TC* Lamivudine, *ZDV* Zidovudine, *NVP* Nevirapine, *EFV* Efavirenz, *WHO* World Health Organization, *TB* Tuberculosis.

### Effectiveness of first-line ART regimens

The effectiveness of first-line regimen among study participants was 83.3% using WHO criteria for virologic failure. Fifteen of the 90 (16.7%) children met the WHO virologic criteria for treatment failure, i.e., HIV RNA of ≥ 5,000 copies/ml after at least 24 weeks on ART [19]. The median time to virologic failure was 7.8 months (range, 5.5 to 27.6 months). Interestingly, only two of the virologic failures met the WHO criteria for immunologic failure. There were neither mortalities nor lost to follow-up during the duration of the study. Three participants switched ARV regimen (3.3%). Two participants switched due to NVP toxicity (generalized rash and unspecified toxicity) and one due to interaction with anti-TB medications. For participants with at least 24 months of follow-up, 71% of these children had undetectable viral loads (HIV RNA <400 copies/ml).

### Longitudinal modeling of CD4 cell count (absolute and percentage) and HIV viral outcomes

Since our setting reflects real world pediatric HIV care where laboratory measures are sparse and usually missing at random (MAR, i.e., the likelihood of an outcome to be missing is not related to the missing data, but can be explained by the observed data), we modeled the CD4 and HIV viral load outcomes. At first available visit, absolute CD4 count ranged between 9 and 3,293 cells/μL, corresponding to 2.2-8.1 on the log scale. CD4 percentage ranged between 0% and 50.5%, and viral load was at a minimum of 63 copies/mL in some children and at a maximum of 3.6 × 10^6^ copies/mL in others.

#### CD4 Absolute Count

**Figure 1:**
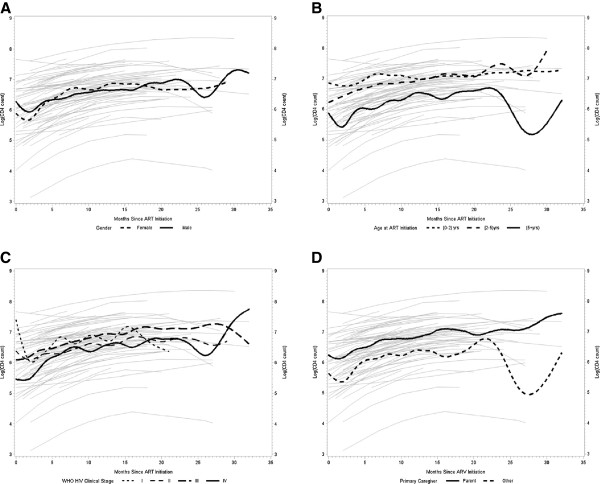
**Correlates of longitudinal changes in CD4 absolute count among HIV-infected children on antiretroviral therapy in Ghana from 2009 to 2012.** CD4 count was log-transformed for statistical modeling. **(A)** Effect of gender on CD4 trajectory. **(B)** Effect of age at ART initiation on CD4 trajectory. **(C)** Effect of WHO Clinical Staging at HIV diagnosis on CD4 trajectory. **(D)** Effect of primary care giver of child on CD4 trajectory.

Table 2 provides a summary of the final model for CD4 cell count. There was a statistically significant increase in the level of CD4 absolute count on the log-scale across time. The increase was not just linear but also quadratic; that is, as the duration of follow-up increased, the CD4 absolute count leveled off. While older age at ART initiation, greater number of available visits during follow-up, female gender, and having more severe WHO HIV clinical staging were all negatively associated with the level of CD4 absolute count on the log-scale, only age at ART initiation reached statistical significance. There were statistical trends for some interactions (Figure 1): (1) females showed a somewhat greater initial slope in the positive change of the outcome over time, but that was mitigated by the negative quadratic slope, resulting in males catching up with girls as the duration of follow-up increased; (2) children with lower WHO HIV clinical staging had on average higher CD4 absolute counts, their rate of increase over time was slower than among children with more severe HIV stage; and (3) children whose primary caretakers were their biological parent(s) had overall greater counts, as well as a higher rate of increase than children who were taken care of by non-biological parents. Of note, there was no statistically significant difference in the levels of CD4 absolute counts with regard to type of ART regimen.

**Table 2:** Model for CD4 count (absolute and percentage)

	CD4 absolute count*, ICC(%) = 0.65		CD4 Percentage*, ICC(%) = 60.5	
**Predictor**	**Parameter Estimate (SE)**	**p-value**	**Parameter Estimate (SE)**	**p-value**
Intercept	7.9 (0.6)	<0.0001	20.7 (5.1)	0.0001
Age at ART Initiation (months)	-0.02 (0.003)	<0.0001	-0.11 (0.01)	0.0004
Number of Available Visits Since ART Initiation	-0.04 (0.04)	0.32	0.93 (0.7)	0.15
Gender				
Female	-0.04 (0.2)	0.85	2.2 (2.0)	0.29
Male (Reference)				
Primary Caregiver				
Parent	0.3 (0.2)	0.07	1.4 (2.2)	0.52
Other (Reference)				
WHO HIV Clinical Stage	-0.2 (0.2)	0.15	-1.5 (1.1)	0.18
ART Therapy				
3TC, ZDV, EFV	0.1 (0.3)	0.66	2.7 (3.0)	0.38
Other	-0.01 (0.4)	0.98	-4.0 (4.9)	0.41
3TC, ZDV, NVP (Reference)				
Time Post Initiation of ART Therapy (months)	-0.10 (0.06)	0.09	3.6 (0.7)	<0.0001
Squared Time Post Initiation of ART Therapy (months)	0.004 (0.002)	0.07	-0.12 (0.03)	0.0003
Interaction Between Time and Age at ART Initiation	0.001 (0.0004)	0.01	-	-
Interaction Between Squared Time and Age at ART Initiation	-0.3^-4^ (0.1^-4^)	0.03	-	-
Interaction Between Time and Female Gender	0.05 (0.03)	0.11	-	-
Interaction Between Squared Time and Female Gender	-0.002 (0.001)	0.06	-	-
Interaction Between Time and WHO HIV Clinical Stage	0.04 (0.02)	0.05	-	-
Interaction Between Squared Time and WHO HIV Clinical Stage	-0.001 (0.0007)	0.09	-	-
Interaction between Time and Number of Visits	-	-	-0.39 (0.11)	0.0003
Interaction Between Squared Time and Number of Visits	-	-	0.01 (0.005)	0.0018

Abbreviations: *ART* Antiretroviral Therapy, *3TC* Lamivudine, *ZDV* Zidovudine, *NVP* Nevirapine, *EFV* Efavirenz, *WHO* World Health Organization.

*Log-transformed for analysis.

#### CD4 Percentages

The only significant changes in CD4 percentage over the follow-up period were attributable to the age at initiation of ART therapy and the number of available follow up visits (Table 2). Similar to the finding for the trajectory of change in CD4 absolute counts, younger age at initiation was associated with overall higher CD4 percentage during the follow-up time, however we did not observe a differential rate of change over time in CD4 percentage due to the age of ART therapy initiation.

#### HIV Viral Load

**Figure 2:**
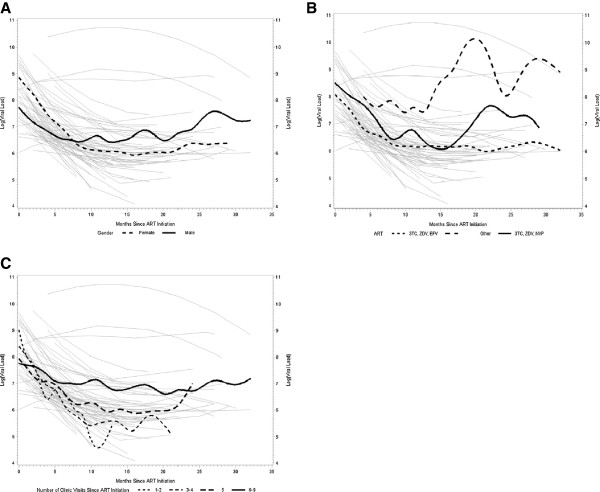
**Correlates of longitudinal changes viral load among HIV-infected children on antiretroviral therapy in Ghana from 2009 to 2012.** Viral load was log-transformed for statistical modeling. **(A)** Effect of gender on viral load trajectory. **(B)** Effect of type of ART regimen on viral load trajectory. **(C)** Effect of number of clinic visits since ART initiation on viral load trajectory.

The rate of decrease in HIV viral load slowed over time. Table 3 provides a summary for this analysis. While females had higher HIV viral loads initially as compared to males, their rate of decline in HIV copies/mL on the log scale was significantly faster (Figure 2A). There was a significant difference in the trajectories of change in the viral load among the ART regimens: compared to 3TC + ZDV + NVP, children on 3TC + ZDV + EFV had on average lower levels of HIV viral load (Figure 2B). Children with 6-8 number of visits had the highest overall level and the slowest rate of decline in viral loads, compared to children with fewer visits (Figure 2C). In general, the slope of decline was steepest over the first 10 months after initiation of treatment.

**Table 3:** Model for viral load, ICC(%) = 19.2

**Predictor**	**Parameter Estimate (SE)**	**p-value**
Intercept	8.1 (1.2)	<0.0001
Age at ART Initiation (months)	0.004 (0.005)	0.45
Number of Available Visits Since ART Initiation	-0.13 (0.16)	0.33
Gender		
Female	1.3 (0.7)	0.06
Male (Reference)		
Primary Caregiver		
Parent	-0.03 (0.4)	0.94
Other (Reference)		
WHO HIV Clinical Stage	0.3 (0.2)	0.15
ART Therapy		
3TC, ZDV, EFV	-1.1 (6)	0.05
Other	-0.5 (0.9)	0.61
3TC, ZDV, NVP (Reference)		
Time Post Initiation of ART Therapy (months)	-0.6 (0.2)	0.002
Squared Time Post Initiation of ART Therapy (months)	0.02 (0.01)	0.02
Interaction Between Time and Female Gender	-0.19 (0.11)	0.08
Interaction Between Squared Time and Female Gender	0.005 (0.004)	0.23
Interaction Between Time and Number of Visits	0.08 (0.03)	0.006
Interaction Between Squared Time and Number of visits	-0.003 (0.001)	0.02

Abbreviations: *ART* Antiretroviral Therapy, *3TC* Lamivudine, *ZDV* Zidovudine, *NVP* Nevirapine, *EFV* Efavirenz, *WHO* World Health Organization.

### HIV drug resistance prevalence and pattern among virologic failures

The median time to virologic failure was 7.8 months. Of the 15 children meeting criteria for virologic failure, sequence results were available for 12 (80%); we were not successful in amplifying DNA from three of the failures. Eight of the 12 children (67%) harbored drug resistant mutations (DRMs) and four (33%) did not harbor any DRMs (Table 4). Sixty-seven percent of children had mutations associated with NRTI, NNRTI, or both NRTI and NNRTI. Of the NRTI-associated DRMs, M184V was the most frequent (8 of 8, 100%). Thymidine-associated mutations (TAMs) were rare with only one patient (1 of 8, 12.5%) harboring T215Y mutation in combination with M184V. Of the NNRTI-associated mutations, K103N (6 of 8, 75%) was the most frequent, followed by Y181C/H (3 of 8, 37.5%). K103N and Y181C were the most frequent NNRTI mutations in patients on EFV- and NVP-based regimens, respectively. M184V and K103N were present in combination in 50% of the patients with DRMs.

**Table 4:** HIV drug resistance prevalence and pattern among HIV-infected children failing HAART in Ghana (N = 12)

**Resistance category**	**Patients, N (%)**
No drug resistance	4 (33)
Resistance to any class	8 (67)
Resistance to NRTI	8 (67)
Resistance to NNRTI	8( 67)
Resistance to NRTI and NNRTI	8 (67)
M184V	8 (67)
T215F/I/S/Y/D	1 (8)
D67N	0
M41L	0
K70R	0
L210W	0
K219E/Q	0
K65R	0
K70E	0
Q151M	0
K101H/E/P/Q	0
K103N	6 (50)
V106M	0
Y181C/H	3 (25)
Y188L	1 (8)
H221Y	1 (8)
P225H	1 (8)
M184V/K103N	6 (50)
M184V/K103N/Y181C/H	1 (8)
M184V/Y181C/H	2 (17)
M184V/T215Y	1 (8)

The second-line regimen widely available in the clinic is 3TC, abacavir (ABC), and lopinavir/ ritonavir. We investigated the susceptibility of the genotypes at virologic failure to second-line regimen. Of the 12 out of the 15 failures with successful genotypic data, susceptibility to 3TC and emtricitabine (FTC) was reduced in 66.7% of the subjects (data not shown). Susceptibility to ABC, the second nucleoside analog of the second-line regimen, was potentially reduced in 75% of subjects. For the other NRTIs (ZDV, d4T, ddT, and TDF), susceptibility was still at 91.7%. All the children were still susceptible to ritonavir-boosted protease inhibitors (PIs).

## Discussion

Our study demonstrates the effectiveness of NNRTI-based first-line regimens used at Korle-Bu Teaching Hospital for HIV-infected children. For 83.3% of the study participants, the first-line ART regimen was effective. At 24 months of follow-up, about 71% of our participants had viral load <400 copies/ml. Both absolute CD4 cell counts and CD4 cell percentages demonstrated a sustained immunologic response through 24 months of follow-up for all participants. Our findings are consistent with reports of effectiveness of ART in HIV-infected children from other countries in sub-Saharan Africa [2-5].

Furthermore, we found high tolerance of the first-line ART regimen in our population based on the low levels of toxicity, and fewer regimen switches. Our rates of regimen switching and toxicity are comparable to previous studies in the region and in other resource-limited settings [4,6]. There were no deaths among the children during the study period. Most importantly, our rates of effectiveness, toxicity, and mortality are comparable to that reported among HIV-infected children in resource-rich countries. Therefore, the unprecedented global effort at scaling up HAART in resource-limited countries is paying off.

The rate of virologic treatment failure after at least 24 weeks was 16.7% among our study participants on their first-line regimen. This is consistent with previously reported rates in pediatric HIV cohorts from the sub-region ranging from 13% to 44% [24-26]. First, consistent with other reports, we found low sensitivity of clinical and immunologic monitoring for detecting virologic treatment failure resulting in HIV drug resistance [27-30]. Interestingly, only two of the virologic failures would have been captured by the WHO criteria for either immunologic or clinical failure – the gold standard for detection of treatment failure [31]. Second, consistent with limited number of studies on early virologic failure in children in sub-Saharan African, the median time to virologic treatment failure in our cohort was 7.8 months (range, 5.5 to 27.6 months) [24,25,30]. Adje-Toure *et al* found that 58% of a cohort of children in Abidjan with detectable viral load after three to five months on therapy harbored HIV DRMs [25]. Therefore, if decisions regarding treatment failure are based solely on clinical or immunologic criteria, most of these children could accumulate multiple DRMs before they are switched to a second-line regimen. Taken together, in the absence of routine virologic testing, HIV-infected children starting ART are at risk for undetectable virologic failure with concomitant development of multiple DRMs that will limit their options for effective second-line regimens.

At virologic failure, 67% of the children harbored viruses with ≥ 1 DRMs, and dual-class resistance was observed in 50% with M84V/K103N being the predominant resistance pathway. Most importantly, our study adds to the limited data on the contribution of early virologic failure to evolution of DRMs in HIV-infected children in sub-Saharan Africa [25]. Furthermore, we observed that the pattern and evolution of resistance mutations is consistent with the components of the first-line regimen as previously reported [24,25,32]. This is valuable information for treatment programs in resource-limited countries in procurement of first- and second-line regimens. Our findings and that of others underscore the urgent need for implementation of viral load monitoring of HIV treatment programs in sub-Saharan Africa to prevent accumulation of DRMs [28,33].

The arguments against routine use of laboratory monitoring in resource-limited settings are sustained by consideration of cost, technical expertise, and lack of infrastructure [34]. While efforts at developing low-cost technologies continue, the question to be answered is: can we adopt a less frequent and targeted testing schedule for CD4 and viral load monitoring? To answer this question, we explored whether readily obtainable data on patients’ characteristics could predict the trajectories of change in CD4 absolute count, CD4 percentage, and HIV viral load. Thus, these predictor variables could inform targeted monitoring strategies. Following ART initiation, we observed a statistically significant increase in CD4 absolute count and CD4 percentage over time. However, the trajectories with time were not linear; they leveled off over time. There was a great degree of variability among children, as expressed by 65% and 61% ICC for CD4 absolute counts and percentages, respectively, supporting the inclusion of the random effect in the model. There were differences in terms of predictors of CD4 absolute counts vs. CD percentages. The negative quadratic slope observed for children with more severe WHO clinical stages suggests a significant slowing in the increase in CD4 absolute counts for that group. This suggests that immune reconstitution may not be robust and complete in severely immuno-compromised HIV-infected children initiating ART. To our surprise, gender and caregiver status played important roles in CD4 absolute count trajectory. Having a biologic parent as a primary caregiver was positively associated with gains in CD4 absolute count. This can be attributed to the benefits of having a stable family with more consistent routines, as compared to losing a parent and having a child’s life disrupted. Female children had better CD4 trajectory. We previously reported that female gender was associated with faster CD4 recovery after initiation of ART [18]. Moreover, a recent Thai study reported that female children had a better immunologic and virologic response than males [35]. There are no reports on association between CD4 recovery or trajectory and gender in pediatrics from sub-Saharan Africa, with the exception of one study that found male gender to be associated with virologic failure [36]. The reasons for this gender effect are not well understood. Interestingly, in HIV-infected adults, gender differences in treatment outcome (i.e., immunologic and virologic) have received mixed reviews [37,38]. The trajectory of change in HIV viral load was a negative reflection of the CD4 longitudinal profile. Of note, female children started out having higher viral loads, but their rate of decrease over time was significantly greater than that of the male children. The role of gender in HIV treatment outcome is very intriguing and needs further investigation.

There are certain limitations to our study. First, this is a single center study and, therefore, one has to be cautious in generalization of our findings. Moreover, the limitations of this analysis include having a relatively small sample size for an observational longitudinal analysis. However, some of the limitations reflect realities in carrying out such a study in a resource-limited setting. At the same time, an important strength of our study is the setting of a real world pediatric HIV clinical care in a resource-limited country with all the perennial laboratory capacity challenges that could represent an overwhelming majority of pediatric HIV clinics in sub-Saharan Africa.

## Conclusions

In conclusion, our study demonstrates that available first-line ART regimens at Korle Bu Teaching Hospital in Accra, Ghana are effective and well tolerated. We also identified important predictors of the trajectories of change in CD4 and viral load after ART initiation, which can inform further studies and feasible targeted laboratory monitoring of ART among HIV-infected children in resource-limited countries. Furthermore, our findings support the scale-up of universal access to the current ART regimens. However, the scale-up should be concurrent with the use of innovative and low cost technologies for laboratory monitoring of ART as the proportion of children with early virologic failure and DRMs is not trivial and is of public health concern. National programs should invest in laboratory capacity to provide at least targeted CD4 and viral load monitoring.

## Competing interests

The authors declare that they have no competing interests.

## Authors’ contributions

OB, JP, EB, LR, VSN, and EP designed the study. OB, JP, EB, MP, WA, JK, BG, and KWCS acquired the data. All authors contributed to the data analysis and drafting of the paper. All authors read and approved the final manuscript.

## Pre-publication history

The pre-publication history for this paper can be accessed here:

http://www.biomedcentral.com/1471-2334/13/476/prepub
